# Bone Marrow T Cells and the Integrated Functions of Recirculating and Tissue-Resident Memory T Cells

**DOI:** 10.3389/fimmu.2016.00051

**Published:** 2016-02-16

**Authors:** Francesca Di Rosa, Thomas Gebhardt

**Affiliations:** ^1^Institute of Molecular Biology and Pathology, Consiglio Nazionale delle Ricerche, c/o Department of Molecular Medicine Sapienza University, Rome, Italy; ^2^Department of Microbiology and Immunology, Peter Doherty Institute for Infection and Immunity, The University of Melbourne, Melbourne, VIC, Australia

**Keywords:** memory T cells, migration, recirculation, bone marrow, tissue-resident T cells, CD8 T cells

## Abstract

Changes in T cell trafficking accompany the naive to memory T cell antigen-driven differentiation, which remains an incompletely defined developmental step. Upon priming, each naive T cell encounters essential signals – i.e., antigen, co-stimuli and cytokines – in a secondary lymphoid organ; nevertheless, its daughter effector and memory T cells recirculate and receive further signals during their migration through various lymphoid and non-lymphoid organs. These additional signals from tissue microenvironments have an impact on immune response features, including T cell effector function, expansion and contraction, memory differentiation, long-term maintenance, and recruitment upon antigenic rechallenge into local and/or systemic responses. The critical role of T cell trafficking in providing efficient T cell memory has long been a focus of interest. It is now well recognized that naive and memory T cells have different migratory pathways, and that memory T cells are heterogeneous with respect to their trafficking. We and others have observed that, long time after priming, memory T cells are preferentially found in certain niches such as the bone marrow (BM) or at the skin/mucosal site of pathogen entry, even in the absence of residual antigen. The different underlying mechanisms and peculiarities of resulting immunity are currently under study. In this review, we summarize key findings on BM and tissue-resident memory (TRM) T cells and revisit some issues in memory T cell maintenance within such niches. Moreover, we discuss BM seeding by memory T cells in the context of migration patterns and protective functions of either recirculating or TRM T cells.

## Introduction

When a pathogen attacks either skin or mucosa, primary immune responses are initiated in the draining lymph nodes (LN) and in some cases in the spleen. In these secondary lymphoid organs, mature antigen-presenting dendritic cells (DC) prime T cells to undergo huge clonal expansion and differentiation into short-lived effector and long-lived memory T cells. Both types of antigen-experienced T cell migrate far from the original priming site, displaying increased capacity to migrate to inflamed tissues as well as to the bone marrow (BM) as compared with naive T cells. Activation-induced changes in T cell membrane expression of chemokine receptors, integrins, and other adhesion molecules underlie this homing behavior. At the end of effective responses, when there is little or no residual antigen left, effector T cells die, leaving behind a small population of long-lived memory T cells, ready to provide protection in case of challenge with the same antigen. Memory T cells can be found all over the body, with a peculiar enrichment either in the BM or at the port of pathogen entry.

We will examine the evidence showing that memory T cells home to the BM and persist over time within this organ, being in constant exchange with blood T cell pool, whereas memory T cells residing at the port of pathogen entry (skin, etc.) are sessile. Moreover, we will review recent data and speculations on the niches wherein either BM or tissue-resident memory (TRM) T cells are maintained over time. We will discuss how BM memory T cells contribute to systemic memory, while TRM cells participate in local protection.

## Bone Marrow Memory T Cells

### A “Reservoir” of Memory T Cells in the BM

The BM consists of islets of hematopoietic BM interspersed with fatty areas, all contained within spongy bone and inside central cavities of long bones. It has long been known that in healthy individuals BM contains mature T cells, which can mediate graft-versus-host disease in T-replete BM transplantation settings. T cells represent about 3–8% of total nucleated BM cells, and have a typically reduced CD4/CD8 T cell ratio, as compared with blood (1, 2). BM T cells include also regulatory CD4 T cells (3). No lymphatic drainage is present, thus BM exchanges with the rest of the body occur only through blood circulation.

Upon T cell priming induced via different routes, T cell contraction is less pronounced in the BM than in the spleen and other organs, and is followed by long-lasting persistence of BM antigen-specific memory T cells (4–9). The BM also contains a high proportion of memory-phenotype T cells, i.e., a heterogeneous subset defined by the expression of activation/memory markers, which increases with aging and includes memory T cells specific for previously encountered antigens (10–12). BM memory T cells contain both central memory (TCM) and effector memory (TEM) T cells, two subsets of recirculating memory T cells identified in blood, having respectively high or low expression of the LN homing receptor CCR7 and distinct homing potential (13–16). Thus, the BM is often described as a “reservoir” for long-lived memory T cells (14, 15, 17).

### Recirculating Memory T Cells in the BM

Pivotal experiments in sheep showed that T cells labeled *in situ* in the BM migrated out of the organ and reached the spleen and other secondary lymphoid organs (18), suggesting that the BM represents a temporary stopping point for recirculating memory T cells (2). In agreement with this notion, parabiosis experiments showed that about 2 weeks after surgery leading to anastomoses of blood vessels between two CD45-congenic mice, comparable numbers of CD45.1^+^ and CD45.2^+^ antigen-specific memory CD8 T cells were found in the BM of each parabiotic mouse (19). Furthermore, intra-vital dynamic imaging studies demonstrated that naive and memory CD8 T cells injected either into the carotid artery or intravenously entered the BM parenchyma of mouse skull and constantly crawled in it (14, 20). Competition among “rival” memory T cells for lodging into the BM was suggested by adoptive transfer experiments showing that memory-phenotype T cells entered BM more easily into young than in thymectomized old mice, where an existing memory T cell pool precluded their free access (11). Such competition with host T cells was lacking when BM T cell recipients were RAG1-deficient mice (21). Thus, it appears that most BM T cells are motile recirculating cells. Some authors argued that the majority if not all of the BM memory T cells are non-migratory cells that permanently inhabit the BM; however, this speculation was based on cell phenotype, activation state, and gene expression analysis (22, 23) and did not take into account the *in vivo* data, including those obtained by *in situ* labeling, parabiosis, intra-vital dynamic imaging, and adoptive transfer (11, 14, 18–20). Nevertheless, the possibility that, similarly to thymus, LN, and spleen (24, 25), the BM also contains a few TRM cells cannot be excluded. For example, parabiosis experiments demonstrated that 3–5% of the antigen-specific memory T cells present in spleen and LN reside permanently in specific locations, i.e., the spleen marginal zone and red pulp and the LN sinuses (25).

In respect to the molecular players of memory T cell homing into the BM, memory CD8 T cells slow down and roll in BM microvessels via L-, P-, and E-selectin-mediated interactions (14). The BM tropism of memory T cells is supported by their high expression of the integrin VLA-4 (α4β1) and strong response to the BM chemokine CXCL12 (11, 14, 26). Conversely, only a few BM CD8 T cells express cutaneous lymphocyte antigen (CLA) and CCR9, involved in T cell homing to skin and gut, respectively (27). CD4 T cells lodge into the BM via molecular mechanisms at least partially similar to those of CD8 T cells. Expression of β1-integrin by CD4 T cells is required for their retention in the BM (28). In addition, CD4 T cell homing to BM is greatly reduced by anti-α2-integrin antibodies (21), suggesting a pivotal role for α2-integrin-mediated interactions, e.g., between the T cell integrin VLA-2 (α2β1) and type I collagen, which is highly abundant in bone. Both CD4 and CD8 T cell localization in the BM was compromised when mice lacked the adhesion molecule VCAM-1 (29).

Molecular regulation of T cell egress from the BM involves Sphingosine-1-phosphate (S1P) interaction with its receptor S1P_1_ (30). S1P levels in the BM are lower than in plasma, so that CD4 and CD8 T cells responding to S1P concentration gradient are normally recruited into the blood, unless S1P_1_ is pharmacologically inhibited by FTY720 (30). In agreement with the inhibition exerted by CD69 on S1P_1_ membrane expression and function (31), it was observed that CD69 ko memory CD4 T cells accumulated in lower numbers in the BM as compared with their WT counterparts (32). However, CD69 deficiency did not cause CD4 T cell increase in blood, implying a more complex scenario (32). Since CD69^+^ cells but not CD69^−^ cells were associated with laminin^+^ stromal cells in the BM, it was proposed that CD69 could mediate retention of memory CD4 T cells within the BM (32). Taken together, these results suggest that CD69 regulates local T cell retention in the BM by a variety of mechanisms.

Certain infectious agents and cytokines can modulate BM T cell exchange with blood, for instance in Human Immunodeficiency Virus (HIV)-infected individuals, CD4 T cells migrate to BM more rapidly than in healthy controls (33). Moreover, after Hematopoietic Stem Cell (HSC)-mobilization procedures, some T cell subsets and especially regulatory T cells increase in the blood, suggesting that they are mobilized from the BM (34, 35). Conversely, high levels of type I IFN induced by acute viral infections block T cell egress from lymphoid organs by a CD69-dependent mechanism (31). Further studies are required to define the rules governing changes in BM T cell recruitment into the blood upon peripheral and systemic immune responses (30, 36).

### Antigen and Cytokines for BM Memory T Cells

Memory T cells specific for previously encountered antigens are commonly found in the BM of healthy subjects as well as of individuals affected by infectious, immune-mediated, and neoplastic diseases. For example, human BM from tetanus toxoid-vaccinated individuals contains tetanus-specific CD4 T cells (22, 37), which can be transferred with BM grafts (37). Pathogen-specific CD4 and CD8 T cells were present not only in the BM of immune individuals after resolution of acute infections (22) but also in the BM of subjects infected by persistent viruses, including Cytomegalovirus (CMV), Epstein–Barr Virus (EBV), and Human Hepatitis C Virus (HCV) (16, 27, 38, 39). BM from neoplastic patients contained tumor antigen-specific T cells, even without intentional immunization (40, 41). Moreover, auto-reactive T cells were found in the BM from individuals affected by autoimmune diseases (42).

In some of these cases, antigens were expressed in the BM, e.g., in EBV-infected subjects (27) or in idiopathic thrombocytopenia purpura patients (42). Though undetected, tumor antigen-positive micro-metastatic cells could be present in patients with solid tumors (41, 43). However, the presence of antigen in the BM was not a reasonable possibility in other cases, for example long time after vaccination against tetanus (22, 37). In rodents, antigen-primed CD4 and CD8 T cell migration and retention into the BM were observed after peripheral immunization procedures that were unlikely to result in the presence of antigen in the BM (7, 44, 45). Hence, it appears that long-lived antigen-specific memory T cells lodge in the BM during immune responses to a variety of antigens, either localized in the BM or not (2).

Furthermore, mouse BM can be a major site of naive CD4 and CD8 T cell priming under conditions of disrupted lymphocyte trafficking in splenectomized mice (46). Blood-borne antigens can reach the BM and induce CD4 and CD8 T cell priming via BM-resident APC (20, 47). Moreover, circulating DC and even neutrophils can transport antigens to BM (48, 49). Altogether, these results show that, under certain circumstances, BM can function as a secondary lymphoid organ.

T cell contacts with other cells in BM niches and molecular interactions influencing T cell activation, proliferation, or survival in BM niches appear heterogeneous. Microscopy studies of BM sections documented that most mouse memory CD4 and CD8 T cells contacted IL-7^+^ VCAM-1^+^ stromal cells (21, 23, 50), whereas some were in proximity to either F4/80^+^ or CD11c^+^ cells (21, 51). Clusters of memory T cells and IL-15-producing cells were observed in human BM sections (52) while a population of 4-1BBL-expressing radioresistant stromal cells supporting memory CD8 T cell survival was described in mouse BM (51). In addition to 4-1BBL, other TNF family members have been involved in T cell survival within BM niches, e.g., GITRL (53–57).

### Acquisition of a BM-Phenotype by Recirculating Memory T Cells

Elegant cellular barcoding experiments in mice clearly demonstrated that after pathogen infection via a systemic route, pathogen-specific CD8 T cells from the BM and those from blood, spleen, and LN all derive from the same set of precursors (58). BM memory T cells exhibit some phenotypic differences when compared with corresponding cells from lymphoid periphery and blood (7, 8, 22, 56, 59, 60). For example, a high proportion of BM memory CD4 and CD8 T cells express CD69 (22, 23, 32). Moreover, memory CD8 T cells from both mouse and human BM have a lower membrane expression of CD127, i.e., the IL-7Rα chain (8, 22, 59, 61), with the exception of antigen-specific CD8 T cells from lymphocytic choriomeningitis virus (LCMV)-infected mice (23). The TNF-R family member GITR is selectively upregulated by a fraction of mouse BM memory CD8 T cells (56). Downregulation of CD127 and upregulation of GITR were both observed in BM but not in spleen samples from WT mice, while they were lost in IL-15 ko mice, suggesting that they are indirect evidence of IL-15 stimulation in BM (56, 60). In agreement with these observations, adoptively transferred splenic CD8 T cells converted to BM-phenotype after entry into the BM (56, 60). Moreover, phospho-STAT-5 and phospho-p38 MAPK were increased in freshly isolated BM CD8 T cells as compared with corresponding spleen cells, possibly reflecting molecular events in the BM, e.g., signaling by IL-15 and TNF family members (59).

No major differences were found by gene-expression analysis of memory T cell paired samples obtained from either BM or blood/spleen, for example mouse BM and spleen CD127^+^ memory CD8 T cells (23) and human BM and blood CD69^−^ memory CD4 T cells (22). This may not be surprising, considering T cell recirculation and the shared ancestry between memory T cells present in BM and in other lymphoid organs (58). After *in vitro* stimulation, memory CD8 T cells freshly isolated from BM strikingly changed their transcriptional profile (23). Of note, BM and spleen memory CD8 T cells had a roughly similar global transcription profile following 2 days of *in vitro* stimulation with anti-CD3^+^ anti-CD28 beads (23), suggesting that the two types of cell shared a common set of genes poised to be expressed after activation (62, 63).

Taken together, these findings support the view that most BM memory T cells cannot be identified as a distinguished subset. They also suggest that BM memory T cells integrate various signals received in the organ, so that their activation state is different from that of recirculating memory T cells from other sources. However, after either egress from the BM or experimental isolation, memory T cells only transiently retain some traits of the stimulation received in the organ (59).

### Contribution of BM T Cells to Memory Maintenance and Secondary Responses

Although the majority of BM memory T cells are quiescent, as demonstrated by staining with the cell cycle marker Ki67 (22, 23), a small percentage of them divides under steady state (8, 9, 60). Indeed, memory T cells have a higher rate of local proliferation in the BM than in spleen and LN, as demonstrated by a variety of experimental approaches (8, 9, 57, 59, 60, 64). For example, by Bromodeoxyuridine, carboxyfluorescein diacetate succinimidyl ester (CFSE), or DNA content assays, BM antigen-specific memory CD8 T cells contained a higher percentage of dividing cells than corresponding cells in spleen, LN, liver, and blood (8, 9). An increased proliferation in the BM was also observed in the case of naive CD8 T cells (59), although as expected their turnover was much lower than that of memory CD8 T cells (65). Estimates of T cell numbers strengthened the view that the BM gives a major contribution to long-term memory T cell maintenance, as well as to the homeostatic regulation of both memory and naive CD8 T cell numbers (2, 8, 9, 66, 67).

CD8 T cell proliferation in the BM is supported by local stimuli, including a dominant role for IL-15, as demonstrated by *in vivo* experiments with IL-15 ko and IL-15Rα ko mice (60). When tested *in vitro*, purified BM CD8 T cells did not exhibit an enhanced proliferative response to IL-7, IL-15, or IL-21 as compared with spleen CD8 T cells (59). Adoptively transferred CD8 T cells originally obtained from either BM or spleen similarly expanded *in vivo* after host injection with Poly I:C, a synthetic dsRNA analog inducing type I IFN and IL-15 (59), as well as upon secondary antigen challenge (68). Taken together, these data support the notion that BM T cells do not exhibit an intrinsic higher proliferative capability, but rather they are constantly stimulated in the BM (59).

Lack of memory T cell lodging into the BM can have a strong impact on memory T cell responses. For example, in CD69 ko mice and in mice treated with anti-α2 integrin antibodies, CD4 T cells were greatly reduced in the BM and help for B cell response was defective (21, 32). Memory CD8 T cells lacking the transcription factor eomesodermin did not populate the BM niche and had impaired long-term persistence and secondary expansion (69). In contrast, β1 integrin-deficient CD4 T cells were not retained in the BM niche but proliferated normally in response to antigenic peptide plus LPS injection (28). Likewise, in the absence of CXCR4, anti-LCMV memory CD8 T cells were defective in migration into the BM, but displayed normal, or even greater, expansion upon secondary challenge with pathogen (70). Further studies are required to clarify these discrepancies, possibly due to pleiotropic effects of key molecule deficiency, and/or differences in the measured aspect of memory response, e.g., T cell self-renewal in the memory phase, T cell expansion upon secondary response, or anti-pathogen protection, after different types of rechallenge.

### BM Memory T Cells in Health and Disease

Bone marrow T cells exert potent antigen-specific effector function, as demonstrated by either adoptive transfers in immunodeficient mice (5) or *in vitro* studies (7). T cells from BM of patients with different types of solid and hematological cancers were able to kill autologous tumor cells (40, 71, 72). Adoptive transfer of BM T cells from breast cancer patients in immunodeficient mice induced regression of xenotransplanted autologous tumors, while blood T cells were not as effective (41). Clinical trials with autologous BM T cells have been initiated in various tumors (73), and the first trial in multiple myeloma patients looks very promising (74).

Moreover, it has been proposed that BM-based T cell proliferation is one of the species-specific advantages allowing Simian Immunodeficiency Virus (SIV)-infected Sooty Mangabeys (SM) but not Rhesus Macaques (RM) to maintain normal CD4 T cell counts (75). Notably, SIV infection does not cause immunodeficiency in SM, whereas it progresses to AIDS in RM, similarly to HIV infection in humans (75).

T cell-derived cytokines can modulate hematopoiesis, implying that BM T cells can contribute to shaping hematopoiesis during both acute and chronic infections (76). For example, IFN-γ released by T cells can either induce IL-6 production by BM stromal cells, in turn augmenting myelopoiesis (77), or also act directly on HSC (78–80).

Bone marrow can play a detrimental role in some T-cell mediated diseases by maintaining pathogenic T cells. In mouse models, pathogenic autoreactive T cells were found in the BM in organ-specific autoimmune diseases, e.g., type I diabetes (81) and chronic uveitis (82). Similarly, in mice with inflammatory colitis, pathogenic CD4 T cells were found in the BM (83). Interestingly, maintenance of pathogenic CD4 T cells required IL-7 in the BM, but not in the colon (84). Thus, it was proposed that, in the disease remission phase, colitogenic CD4 T cells persisted in the BM (83, 84).

Moreover, T cell effector function in the BM can stimulate pathological bone resorption, by activating osteoclasts. It is well established that CD4 T cells recruited in joints and periodontal tissue of patients affected by rheumatoid arthritis and periodontitis, respectively, stimulate osteoclastogenesis by producing IL-17 and RANK-L (85–88). Recently, a subset of osteoclastogenic Th17 TNF-α producing cells has been identified in PBMC from patients with Crohn’s disease, and it has been proposed that these cells can migrate to the BM and mediate bone loss, in agreement with mouse models (89–91). Notably, in a mouse model of breast cancer, pro-osteoclastogenic BM T cells favored the establishment of skeletal metastases by inducing osteolytic lesions (92).

Finally, T cells regulate physiological processes occurring in the BM, i.e., normal hematopoiesis and bone tissue homeostasis. Surprisingly, the maintenance of normal bone mass and bone mineral density in physiological conditions is promoted by T cells, which stimulate the production of the RANK-L decoy receptor osteoprotegerin by B cells, through CD40L/CD40 interaction (93). A cross-talk between T cells and hematopoietic precursors occurs in the BM in normal healthy conditions (94, 95). For example, it has been shown that BM T cells sustain normal granulopoiesis (94), while regulatory T cells inhibit excessive T cell-production of the granulopoiesis-promoting cytokines GM-CSF, TNF, and IL-6, thus allowing for sufficient B lymphopoiesis (95). Regulatory T cells in the BM are required for HSC engraftment upon transplantation (96, 97), and likewise might protect normal HSC and their niches from destructive immune responses (97). Taken together, these results suggest that BM T cells are engaged in a complex interplay with other cells in the local environment, contributing to maintain bone and BM integrity and function.

## Tissue-Resident Memory T Cells

### A “Reservoir” of Memory T Cells in Non-Lymphoid Tissues

In addition to the BM and secondary lymphoid organs, the body’s surfaces such as the linings of the skin, gut, and reproductive tract also harbor large numbers of CD4 and CD8 T cells (6, 98–100). Most of these peripheral T cells are antigen-experienced memory cells and are generally believed to provide specific immunity against renewed infection with previously encountered pathogens. Given their location in close proximity to the external environment, it appears likely that some of these memory T cells also recognize commensal microbiota, and such T cell–microbiota interactions have been proposed to fine-tune peripheral immunity (101, 102).

While it is clear that T cells recirculate between peripheral tissues and the blood via the lymphatic system (103–105), there is recent evidence for a non-recirculating population of memory T cells that remain localized to peripheral tissues and never return to the blood (106, 107). Such TRM cells are best characterized for the CD8 subset and have been described in a large number of peripheral organs, including skin, gut, brain, salivary glands, lungs, female reproductive tract, and others (106, 107). Furthermore, non-recirculating memory T cells also exist in lymphoid organs such as LN and thymus (24, 25).

### Sessile TRM Cells Permanently Residing in Tissues

Various experimental strategies, such as organ transplantation, sex-mismatched adoptive T cell transfer, and parabiosis in mice have unequivocally demonstrated that TRM cells can persist in peripheral tissues in disconnection from the pool of recirculating T cells in the blood (108–111). In line with this, CD8 TRM cells are often found in specialized microanatomical compartments such as the epithelial layers of skin and gut where they are sequestered from direct lymphatic drainage by the underlying epithelial basement membrane.

These epithelial CD8 TRM cells commonly express surface receptors such as CD103 (the αE integrin subunit), CD69, and the integrin VLA-1 (α1β1), which are variously involved in T cell retention and persistence (107, 112). For instance, genetic deficiency in CD103 expression results in defective generation of CD8 TRM cells in gut mucosa (113, 114), abolished accumulation of CD8 T cells in islet grafts (115), as well as a gradual loss of TRM cells from skin and lung mucosa (116, 117). Similarly, genetic or functional ablation of VLA-1 causes a dramatic decline in memory CD8 T cell numbers in lung after pulmonary virus infection (118). While these adhesion molecules are likely to mediate the tethering of TRM cells to their microenvironment, integrin binding and ligation may also support T cell survival and functional activity (119, 120). By contrast, the surface molecule CD69 can act to block the functional activity of the tissue exit receptor, S1P_1_, and thereby inhibit tissue egress of effector T cells with the potential to undergo local differentiation into TRM cells (31, 121). In addition to this posttranslational regulation of S1P_1_ function, the progressive transcriptional downregulation of S1P_1_ expression appears to be a critical checkpoint in the TRM maturation pathway in a variety of tissues (117, 122). The high level surface expression of CD69 by fully developed TRM cells further echoes the absence of S1P_1_ expression, rather than recent T cell receptor activation, as both molecules physically interact with each other, resulting in mutual inhibition of surface expression (123).

In addition to epithelial CD103^+^CD8^+^ TRM cells, phenotypically and anatomically distinct CD103^−^ CD8^+^ TRM cells, including those that form clusters at sites of intestinal infection with *Yersinia pseudotuberculosis*, have also been described more recently (100, 124). Likewise, presumably non-recirculating or long-term retained memory T cells have been identified among CD4^+^ memory T cells in a variety of tissues, including skin, as well as lung, gut, and vaginal mucosa (125–129). Some of these also express markers initially described for CD8^+^ TRM cells, such as CD69 and CD103, although the role of these molecules in tissue persistence of CD4^+^ TRM cells remains largely unknown. It is therefore likely that TRM cells exist among both CD4^+^ and CD8^+^ memory T cells, and future studies will have to compare the molecular and functional relationship between those different types of TRM cells. Nevertheless, given that the molecular pathways underpinning the maturation and persistence of TRM cells have so far most extensively been studied for CD103^+^ CD8^+^ TRM cells, we will focus our attention on this subset for the remainder of this article.

### Antigen and Cytokines for CD103^+^ CD8^+^ TRM Cells

The highest frequencies of CD8^+^CD103^+^ TRM cells are found in previously infected or inflamed tissue (108, 110, 130), where TRM cells develop from infiltrating effector T cells during lesion resolution (114, 117). Accordingly, peripheral infection can be regarded as the principle inducer of TRM cells in barrier organs such as skin and mucosa. In addition, systemic infections that generate a large pool of memory precursor T cells disseminating throughout the body can give rise to TRM populations in a variety of barrier tissues and internal organs (6, 111, 113, 131). Likewise, repeated immunization has been shown to progressively build up considerable numbers of TRM cells even in non-infected areas of skin (110).

Tissue-resident memory precursor cells are found within the KLRG1^−^ effector population that also contains the precursors of circulating memory CD8 T cells (114, 117, 132). Of note, TRM precursor effector cells rapidly cease to migrate through peripheral tissues after pathogen clearance (111, 130), meaning that the pool of TRM cells is established early after infection and remains stable with minimal external input thereafter.

The generation of TRM cells in infected skin is regulated by a complex interplay of processes such as peripheral infiltration, tissue exit, local positioning, and survival, which ultimately results in the maturation of a long-lived population of TRM cells in the epidermal layer. Indeed, epithelial infiltration is critical for TRM formation, at least in skin, since effector T cells that are prevented from epidermal entry by pertussis toxin treatment fail to generate long-lived CD103^+^ memory cells (117). This compartment-specific generation of T cell memory in skin likely reflects the anatomical restriction of essential differentiation and survival factors such as TGF-β and IL-15 that are expressed at high levels in the epithelial layer. Further supporting this assumption, CD8 T cells fail to acquire a CD103^+^ TRM phenotype and are lost from skin over time in absence of TGF-β and IL-15 signaling (117).

In addition to these roles of TGF-β and IL-15 in TRM differentiation and survival in skin, the same or other essential cytokines and local factors may be involved in the establishment of TRM populations in an organ- or location-specific manner (113, 114, 116). Local antigen recognition for instance is strictly required for the establishment of long-lived TRM populations in organs such as brain, sensory ganglia, and lungs (120, 130, 133, 134). By contrast, in many other peripheral tissues and internal organs, including skin, gut, kidneys, and salivary glands, local antigen recognition appears to be dispensable for TRM development (113, 130). Nevertheless, it is conceivable that local antigen stimulation enhances TRM accumulation, possibly through the downregulation of tissue exit receptors such as CCR7 or through direct effects on local proliferation and survival of TRM precursor cells.

### Tissue-Specific Gene Expression Signature of CD103^+^CD8^+^ TRM Cells

Importantly, local memory differentiation by tissue infiltrating effector T cells is accompanied by the progressive acquisition of a specific transcriptional program that distinguishes TRM cells from their TEM and TCM counterparts in the circulation (117, 122). Interestingly, the majority of these TRM-specific transcripts appear to be imprinted by the organ of residency. Skin CD8 TRM cells for instance express CCR8 whereas those in gut mucosa express CCR9 (130), and both chemokine receptors have been implicated in organ-specific homing to the respective anatomical locations. In addition, approximately a quarter of the TRM-specific transcripts are commonly up or downregulated in different CD103^+^ TRM cell populations from skin, gut, and lungs when compared to recirculating memory cells. This TRM-specific core transcriptional profile across a variety of organs comprises genes encoding for migration and adhesion molecules (e.g., *Itga, Itga1*, and *S1pr1*), transcription factors (e.g., *Eomes*, *Hobit*, and *Litaf*), immunoregulatory molecules (e.g., *Cd244*, *Ctla-4*, and *Icos*), and various enzymes (e.g., *Inapp4b, Cmah*, and *Qpct*) and likely echoes a common molecular program underpinning the formation, persistence, and function of CD103^+^ CD8 TRM cells in peripheral tissues (130).

Future work will have to define how stably this transcriptional profile is imprinted in TRM cells, and whether TRM cells can adopt alternative transcriptional and functional programs on renewed antigenic challenge or experimental release from the tissue context. Relevant to this, it will be important to test whether the same epigenetic modifications that regulate chromatin accessibility and gene transcriptional programs in recirculating memory T cells (62, 63) also operate in TRM cells. Likewise, the extent to which the CD8 TRM transcriptional signatures and their putative epigenetic regulators overlap with those in CD103^−^ and/or CD4 TRM cells is currently unknown and needs to be tested. Regardless, there is accumulating evidence that a considerable proportion of T cells in peripheral non-lymphoid tissues adopt a phenotype and transcriptional profile that clearly distinguishes them from recirculating memory T cells.

### Contribution of CD103^+^ CD8^+^ TRM Cells to Local Immunity

Owing to their strategic location at body surfaces, TRM cells can immediately sense invading pathogens and initiate a rapid local immune response (112). In skin, for instance, TRM cells use dendritic protrusions to probe their environment, a behavior that reflects their active contribution to skin immune surveillance (109, 135). Following activation by their pathogen-specific T cell receptors, TRM cells produce a number of proinflammatory mediators, including the cytokines IFN-γ, TNF-α, and IL-2 (112, 136–139). These mediators drive the local activation and recruitment of innate and adaptive immune cells and also act on stromal and parenchymal cells to confer a resistance to pathogen dissemination (112, 136, 138, 139). In addition, TRM cell may directly lyse infected target cells, although the contribution of cytotoxicity in TRM-mediated protection remains to be demonstrated.

Tissue-resident memory cells have been shown to provide an exceptional level of immediate protection from renewed infection with a broad variety of viral and bacterial pathogens (108–110, 114, 134, 135, 138, 140). Furthermore, TRM cells generated by immunization strategies combining T cell priming with local treatment with inflammatory adjuvants provide a level of protection from *de novo* infection in skin and mucosa that is far superior to what can be achieved by circulating memory cells (130). Likewise, local immunizations with human papilloma virus vectors or treatment with T cell-attracting chemokines have been shown to generate protective TRM cells in vaginal mucosa and the female reproductive tract (141, 142).

Since these experiments confirm that TRM cells do not rely on antigen for long-term persistence, unlike other approaches used to generate T-cell immunity in peripheral tissues (143), TRM cells are now regarded as promising mediators of long-lived peripheral immunity for future vaccines (144). Importantly in this respect, a series of landmark papers have demonstrated the existence of Herpes Simplex Virus (HSV)-specific CD8^+^ memory T cells resident at the dermal-epidermal junction in human genital skin (145–147). Remarkably, these resident T cells share transcriptional commonalities with HSV-specific TRM cells in mice (148) and are involved in preventing genital lesions upon asymptomatic HSV-2 shedding in skin (147). Furthermore, CD8^+^ memory T cells with a CD103^+^CD69^+^VLA-1^+^ TRM phenotype also exist in other human tissues (129, 149, 150). Such observations strongly argue that TRM cells are critical mediators of peripheral immunity in both mice and humans, and it is has been speculated that one of their principle functions is in dealing with recurrent or persistent infections in defined anatomical niches such as epithelial or neuronal compartments (151).

While in such cases, TRM cells exert highly beneficial protective functions, pathogenic TRM responses may also cause tissue pathology. Supporting this notion, accumulation and aberrant activation of TRM cells have been described in localized and recurrent human diseases such as skin autoimmunity and transplant rejection (152). Early studies using xenotransplantation of pre-psoriatic human skin onto mice, for instance, have demonstrated that graft-derived human TRM cells, in absence of circulating memory T cells, are sufficient to drive development of psoriasis lesions through localized production of inflammatory cytokines (153, 154). Similarly, IFN-γ producing epidermal TRM cells can initiate skin lesions upon ingestion of drugs that cause recurrent fixed drug eruptions (155), and CD8^+^ T cells with a TRM phenotype have been described in alopecia and vitiligo lesions (156, 157). Another impressive demonstration of TRM-mediated pathology in humans stems from a recent study that defined donor-derived TRM cells as the major drivers of localized rejection responses after face transplantation (158). Finally, CD103^+^CD8^+^ T cells make up a considerable proportion of tumor-infiltrating lymphocytes in carcinomas and brain tumors, and their peri-tumoural accumulation is associated with improved clinical outcomes (159–162).

In summary, an accumulating body of literature implies a critical role of protective as well as pathogenic TRM responses in infectious and inflammatory diseases in humans. While so far this has most intensively been studied in easily accessible organs such as skin, it is tempting to speculate that TRM cells act as central mediators of localized diseases also in lungs, gut, and many other internal organs. Future studies will have to further clarify the role of protective and pathogenic TRM cell responses in humans and will have to explore avenues for their prophylactic or therapeutic manipulation in experimental and clinical settings.

## Concluding Remarks

The postulated division of labor between recirculating memory T cells and TRM cells offers a novel view of both memory maintenance and response to antigenic rechallenge that integrates and broadens the previous perspective based on TCM/TEM paradigm (13).

**Figure 1:**
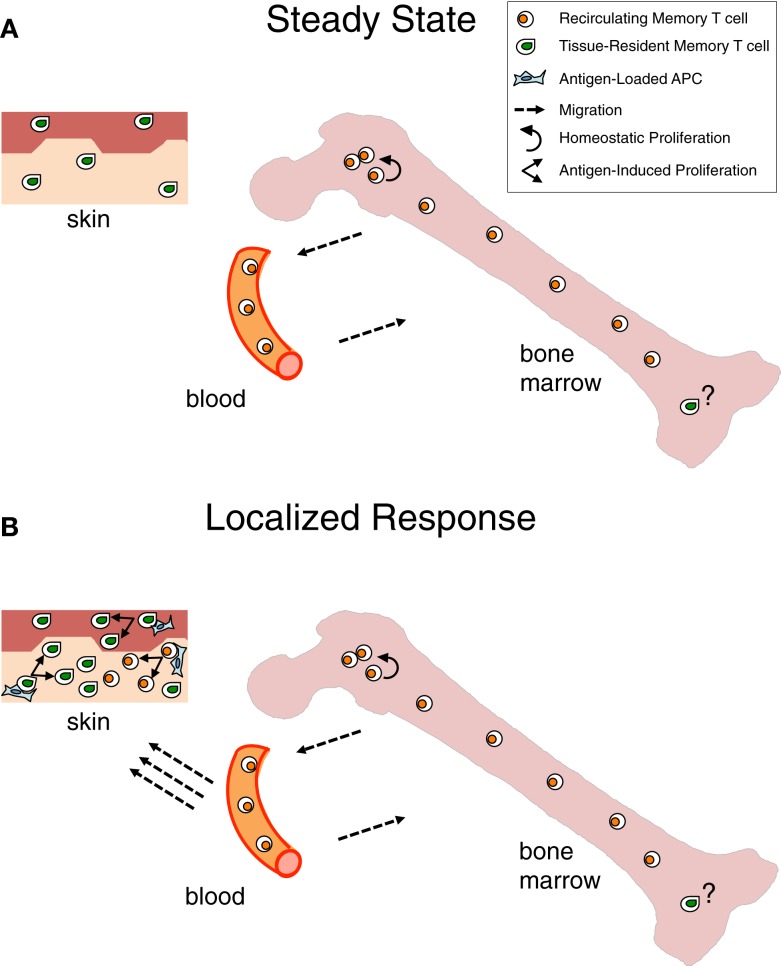
**Contribution of bone marrow memory T cells and tissue-resident memory T cells to long-lasting immunity**. **(A)** Under steady state, Bone Marrow (BM) memory T cells are in constant exchange with the recirculating memory T cell pool, while tissue resident memory T (TRM) cells are fixed within peripheral organs, e.g., skin, in a quiescent state. Although the majority of BM memory T cells are quiescent, a minor proportion of them self-renew within BM niches, mostly in response to cytokines, e.g., IL-15. This homeostatic proliferation counterbalances losses due to cell death and contributes to maintain constant numbers of recirculating memory T cells over time (8, 9). The putative possibility that a few TRM cells exist in the BM is depicted. **(B)** When infection is localized in peripheral organs, TRM cells represent a first line of defense that is reinforced by the arrival of recirculating memory T cells, acting as a second line of protection. Antigen presented by APC in the peripheral tissue leads to immediate antigen-specific expansion and effector response of the TRM and, with some delay, of the newly recruited memory T cells (163, 164).

Long-lived persistence of memory T cells is achieved in the steady state by different mechanisms. Recirculating memory T cells rely on a finely tuned equilibrium between quiescence and homeostatic proliferation, which is mostly achieved within the BM niches wherein these cells temporarily stop. In contrast, TRM cells live permanently as sessile non-migratory cells within skin/mucosal niches, wherein they survive in a quiescent state (Figure 1A).

It appears that recirculating memory T cells and TRM cells provide respectively systemic immunity and immediate protection at the port of pathogen entry. Yet, the two types of memory T cells act in concert for tissue protection as recirculating memory T cells are recruited to skin or mucosal sites of secondary challenge, resulting in both more efficient local effector response and boosting of systemic memory (Figure 1B). Naturally, recirculating memory T cells arrive with some delay in the tissue, while TRM cells are already there for highly efficient immediate protection.

**Figure 2:**
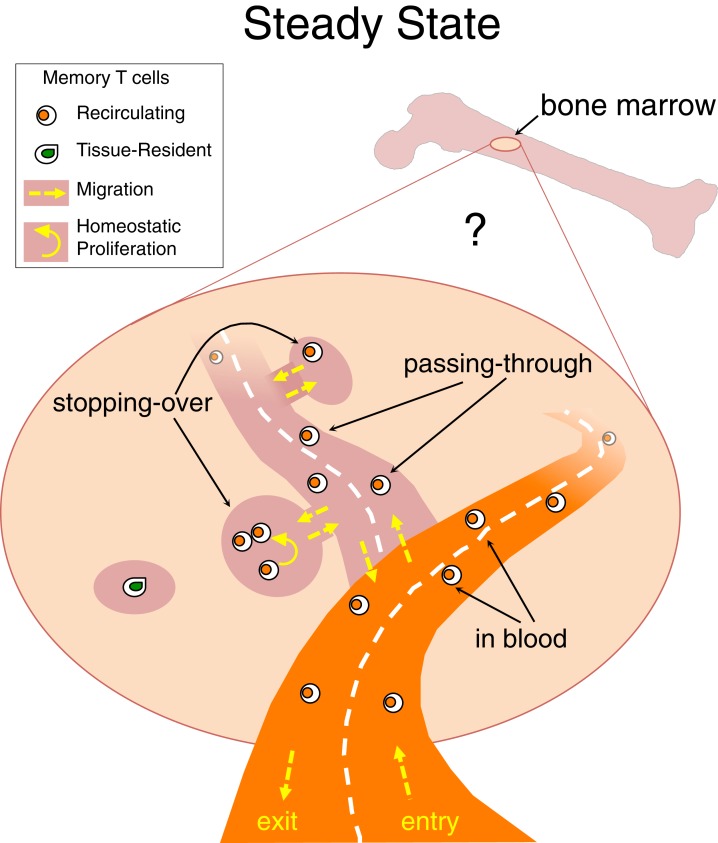
**Stopping-over, passing-through and tissue-resident memory T cells in bone marrow**. Under steady state, memory T cells migrate into the BM and then circulate back to the blood, with poorly defined kinetics. It is possible that some recirculating memory T cells quickly transit through the BM parenchyma while others stop over for some time within BM niches. A few memory T cells might stay permanently in BM niches and never return to the blood, representing the equivalent of tissue-resident memory (TRM) T cells identified in other organs.

Taking into consideration that TRM cells have been identified not only at the epithelial barriers of the body but also in lymphoid and/or internal organs, e.g., LN and brain (106, 107), it is likely that each organ harbors some TRM cells and that BM is not an exception to this rule (Figure 1). The peculiarity of the BM would be that under steady state most memory T cells in it belong to the recirculating pool. Some heterogeneity might exist in the kinetics of recirculating memory T cell transit into the BM, with some cells just quickly passing through and others stopping over during their journey, thus inhabiting for some time the BM niches wherein they receive signals for survival/homeostatic proliferation (Figure 2).

Despite their different migration pathways, positioning and role in immunity, both TRM and BM T cells participate in a local network of cellular and molecular interactions in the organ where they are located, influencing normal tissue homeostasis and organ function. In the case of BM T cells, it has been shown that they normally regulate hematopoiesis, as well as bone metabolism. In respect with TRM cells located in barrier organs, it is conceivable that they protect host’s health and sometimes contribute to disease in several manners, for example they might shape the gut microbiota composition, with a possible indirect impact on metabolic syndrome, obesity-related disorders, inflammatory bowel disease, and colorectal cancer (101, 165). Some of the unsolved questions on BM memory T cells and TRM cells are listed in Box 1.

Box 1 **Unsolved questions on bone marrow (BM) memory T cells and tissue-resident memory (TRM) T cells.**

*Tissue-specific signals for differentiation*: After naive T cell priming, what are the distinct signals regulating differentiation of either recirculating memory T cells or TRM cells? Do recirculating memory T cells integrate signals for differentiation they receive over time in diverse environments (e.g., within BM, spleen, and LN)? Do TRM cells lodged in diverse tissues receive in each location a specific combination of signals for differentiation?

*Tissue-specific signals for survival/homeostatic proliferation*: What are the external signals regulating the long-lasting maintenance of recirculating memory T cells within BM niches and/or TRM cells within tissue niches? What is the role of external signals (including antigen, IL-15, TGF-β, etc.) in the quiescence/self-renewal of BM memory T cells and/or in the survival in a quiescent state of TRM cells?

*Heterogeneity*: If TRM cells receive a specific combination of signals for differentiation and maintenance in each organ or location, and if recirculating memory T cells integrate diverse signals over time, are TRM cells, taken as a whole, expected to be more heterogeneous than recirculating memory T cells? Is it conceivable that several different subsets of location-specific TRM cells do exist?

*Intracellular networks orchestrating differentiation*: What are the intracellular molecular pathways regulating differentiation and stable genetic imprinting or plasticity of TRM and recirculating memory T cells? How do key intracellular molecules regulate epigenetic marking, gene transcription, protein translation, metabolic state of TRM and of recirculating memory T cells?

*Regulation of absolute cell numbers*: Is homeostatic maintenance of memory T cell numbers independently regulated for TRM cells and for recirculating memory T cells (including those in BM, spleen, and LN)?

*CD4 and CD8 T cells*: Do TRM cells resemble recirculating memory T cells in respect to the shared and/or specific protective functions of the CD4 and the CD8 T cell subsets? Are differentiation and maintenance of the CD4 TRM similar to those of the CD8 TRM?

*Aging*: Are there changes occurring with aging in the distribution of tasks between TRM and recirculating memory T cells?

*Normal tissue homeostasis and organ function*: Do BM memory T cells and TRM cells play a role in maintaining normal tissue homeostasis and organ function?

## Author Contributions

FR conceptualized the article, prepared the box and the figures, and wrote the section on BM T cells; TG wrote the section on TRM cells; FR and TG worked together on the remaining sections.

## Conflict of Interest Statement

The authors declare that the research was conducted in the absence of any commercial or financial relationships that could be construed as a potential conflict of interest.
